# End-to-End Privacy-Aware Federated Learning for Wearable Health Devices via Encrypted Aggregation in Programmable Networks

**DOI:** 10.3390/s25227023

**Published:** 2025-11-17

**Authors:** Huzaif Khan, Rahul Kavati, Sriven Srilakshmi Pulkaram, Ali Jalooli

**Affiliations:** Department of Computer Science, California State University Dominguez Hills, 1000 E. Victoria Street, Carson, CA 90747, USA; hkhan17@toromail.csudh.edu (H.K.); rkavati1@toromail.csudh.edu (R.K.); spulkaram1@toromail.csudh.edu (S.S.P.)

**Keywords:** federated learning, homomorphic encryption, in-network computing, edge computing, graph neural networks, healthcare IoT

## Abstract

The widespread use of wearable Internet of Things (IoT) devices has transformed modern healthcare through the real-time monitoring of physiological signals. However, real- time responsiveness and data privacy are big challenges. Federated Learning (FL) keeps direct data exposure to a minimum but is susceptible to inference attacks on model updates and heavy communication overhead. In-network computing (INC) solutions currently offer greater efficiency but without cryptographic security, whereas homomorphic encryption (HE) offers high privacy but at the expense of latency and scalability. To bridge this gap, we present Edge-Assisted Homomorphic Federated Learning (EAH-FL), a framework that unifies Cheon–Kim–Kim–Song (CKKS) HE with in-network aggregation. Lightweight clients outsource encryption and decryption to trusted edge devices, whereas programmable switches carry out aggregation in the encrypted domain. Massive-scale simulations over realistic healthcare data sets demonstrate that EAH-FL preserves near-plaintext model accuracy (F1-score > 0.93), delivers packet delivery ratios > 0.95, and converges well for various client scales. The encryption expense is mostly incurred by the edge layer rather than resource-constrained wearables. Through the use of encryption, in- network acceleration, and smart routing, EAH-FL provides the first practical solution that achieves strong privacy, low latency, and scalability for real-time federated learning in healthcare in a single solution. These results validate its viability as a deployable and secure building block for next-generation digital health monitoring.

## 1. Introduction

The rapid adoption of wearable Internet of Things (IoT) devices has transformed modern healthcare by enabling continuous, real-time monitoring of physiological signals such as heart rate, oxygen saturation, blood pressure, and physical activity. This constant stream of multimodal physiological data supports predictive diagnostics, chronic disease management, and timely clinical interventions, significantly improving patient outcomes and advancing personalized medicine. While the ubiquity, continuity, and personalization of health IoT make it highly appealing, these same attributes also introduce critical challenges related to data privacy, latency, communication overhead, and adherence to stringent regulations such as Health Insurance Portability and Accountability Act (HIPAA) and the General Data Protection Regulation (GDPR). In latency-sensitive scenarios such as arrhythmia detection or hypoxemia alerts, even a delay of a few seconds can mean the difference between life and death, underscoring the criticality of low-latency and privacy-preserving learning frameworks. Despite increasing attention, healthcare remains a uniquely challenging domain for the deployment of FL. Unlike consumer-facing applications such as keyboard prediction or recommendation systems, healthcare IoT data are inherently high-stakes and sensitive. Any latency in processing vital signs can delay life-critical interventions, and any breach of confidentiality can result in irreparable harm to patient trust, regulatory violations, and substantial financial penalties. A 2025 report [1] by IBM estimated that the average cost of a healthcare data breach exceeded $9.77 million. Thus, frameworks designed for medical IoT cannot simply inherit generic FL or HE mechanisms they must meet more stringent requirements for latency, security, and scalability simultaneously Traditional cloud-centric machine learning architectures are increasingly inadequate for such stringent healthcare requirements. These centralized approaches require transmitting raw physiological data to remote servers for model training, creating bottlenecks in both latency and bandwidth while exposing sensitive health information to potential privacy breaches. FL has emerged as a promising paradigm that enables distributed devices to collaboratively train a shared model without exchanging raw data, thereby reducing privacy risks and facilitating regulatory compliance. While baseline algorithms such as FedAvg and its extensions (e.g., FedProx and FedDyn) address client drift to some extent, studies show that these approaches remain sensitive to healthcare data heterogeneity and lack robust privacy guarantees [2,3].

To enhance confidentiality during decentralized learning, researchers have incorporated HE into FL to perform computations on encrypted model updates. Works such as FedNIC [4], Shen et al. [5], and Walskaar et al. [6] demonstrate secure aggregation using single and multi-key HE schemes, ensuring gradients remain private throughout the training process. However, these solutions introduce high computational and communication costs, rendering them impractical for resource-constrained IoT devices. Other studies have explored blockchain-assisted HE to improve trust and accountability [7,8], yet they still suffer from scalability and latency bottlenecks. More recent research by Gowri et al. [9] and Khan et al. [10] applied the Cheon–Kim–Kim–Song (CKKS) approximate HE scheme in medical and edge-AI scenarios, respectively, achieving comparable accuracy on encrypted data while significantly reducing execution time. These findings suggest that lightweight, edge-enabled encryption is feasible, but the integration of privacy and efficiency within a unified framework remains an open challenge.

In parallel, In-Network Computing (INC) has gained importance as an effective paradigm for reducing communication delays and bandwidth consumption by enabling programmable network devices to perform intermediate model aggregation.Approaches such as FediAC [11], GAIN [12], and FLIP4 [13] demonstrate substantial throughput and convergence improvements by enabling near-data aggregation. Nevertheless, These INC frameworks operate primarily on plaintext model updates and therefore cannot ensure data confidentiality a critical limitation in regulated medical applications. Recent surveys by Caruccio et al. [14] and Albshaier et al. [15] emphasize that achieving true privacy-preserving and low-latency FL requires the joint integration of cryptographic protection and system-level optimization through edge-assisted architectures.

Motivated by these limitations, this study proposes the Edge-Assisted Homomorphic Federated Learning (EAH-FL) framework, which integrates HE with in-network aggregation to achieve both privacy and efficiency in healthcare IoT environments. EAH-FL employs selective encryption to reduce cryptographic overhead and utilizes trusted edge servers to offload aggregation tasks on encrypted data, maintaining end-to-end confidentiality while minimizing latency. Experimental evaluations show that EAH-FL reduces communication latency by up to 46% and encryption overhead by 38% compared to conventional HE-FL methods. The marginal accuracy loss is less than 1.2% in non-IID healthcare datasets. Furthermore, EAH-FL enhances throughput and energy efficiency in multi-client edge environments, demonstrating its potential for real-time, regulation-compliant healthcare analytics.

The main contributions of this paper can be summarized as follows:

- A formally defined mathematical framework for encrypted edge-assisted federated learning. Unlike prior heuristic or architecture-only FL designs, EAH-FL provides a rigorous mathematical formulation that models client updates, encrypted aggregation, and edge-level optimization under HE. This formalism allows analytical reasoning about latency, encryption cost, and convergence behavior.
- A novel hybrid architecture with HE+INC and EAH-FL. This framework jointly leverages programmable edge nodes for in-network encrypted aggregation, ensuring that all model updates remain encrypted end-to-end. This integration bridges the gap between privacy-preserving cryptography and system-level acceleration achieving both confidentiality and real-time performance.
- The Grahical neural network (GNN)-assisted topology inference module improves encrypted model aggregation efficiency by learning dynamic inter-client relationships. Experimental results show up to 23% lower latency, 31% communication reduction (CRR), and a 12% improvement in convergence stability compared to static edge aggregation.
- Comprehensive empirical validation and analysis. Extensive experiments including baseline comparisons, ablation studies, and parameter sensitivity analyzes confirm that EAH-FL achieves a superior balance of privacy, efficiency, and scalability compared to state-of-the-art FL frameworks. The proposed model outperforms HE-only and INC-only systems, proving its feasibility for real-time healthcare IoT environments.

Overall, the proposed framework bridges the gap between privacy preservation and operational efficiency in federated healthcare systems, demonstrating that secure, low-latency learning is achievable through the synergy of edge computing and HE.

## 2. Related Work

FL has become a cornerstone for privacy-preserving machine learning in domains where data sensitivity and regulation prevent centralized aggregation. Early studies established FedAvg as the practical baseline. Lee et al. [3] reviewed the stability of FedAvg under various conditions, including medical image classification tasks, and found that it maintained stable convergence even when the data distributions were highly heterogeneous. This reinforced its role as a strong baseline, though it also highlighted that FedAvg alone does not handle all the challenges of healthcare data heterogeneity. More advanced methods, such as FedProx and FedDyn, were developed to mitigate client drift under non IID data. While FedProx constrains local updates through a proximal penalty, and FedDyn dynamically corrects local objectives, Yang et al. [2] showed that even with these techniques, FL in medical imaging remained sensitive to non-IID data and still required additional privacy-preserving mechanisms. Recent studies have reinforced this dual requirement. Mao et al. [8] proposed EPFed, a blockchain-assisted FL framework integrating HE and secret sharing to balance privacy and computational efficiency. Likewise, Aziz et al. [16] reviewed the intersection of Differential Privacy (DP) and HE for FL, concluding that hybrid cryptographic frameworks can mitigate gradient leakage while maintaining convergence stability. These insights underscore the need for privacy-preserving frameworks that simultaneously optimize latency and communication efficiency in real-world healthcare environments.

A significant line of research focuses on enhancing privacy through HE. Choi et al. [4] introduced FedNIC, which offloads HE operations to SmartNICs, thereby reducing server-side computation overhead and improving efficiency. However, their work still relies on centralized aggregation, which introduces latency and scalability bottlenecks. Yang et al. [2] proposed a dynamic corrected split FL framework for medical image networks, applying HE to protect updates in specific layers. Although effective for U-shaped models in imaging, their approach limited encryption to selected layers and did not reduce communication costs. Shen et al. [5] tackled encryption flexibility by presenting a multi-key HE framework that allows different clients to encrypt with distinct keys while still supporting secure aggregation. Similarly, Walskaar et al. [6] demonstrated a practical medical FL system using the Flower framework combined with multi-key FHE, confirming feasibility but also exposing performance issues due to high cryptographic overhead. Naresh and Varma [17] applied HE in a healthcare-specific case of heart stroke detection, showing that HE-enabled FL could support clinical decision-making, but they did not address latency or energy efficiency in IoT devices. Firdaus et al. [7] combined blockchain with HE to improve accountability in healthcare data sharing. While this strengthened trust, their results showed high encryption overheads and limited scalability for latency-sensitive environments. Collectively, these works confirm the strength of HE for confidentiality but show that HE alone is often too heavy for real-time healthcare IoT deployments. Recent implementations have begun to address this limitation. Gowri et al. [9] employed the CKKS approximate HE scheme for secure medical diagnostics, achieving over 70% model accuracy on encrypted data while preserving patient privacy. Similarly, Khan et al. [10] demonstrated the feasibility of CKKS-based full HE on resource-constrained edge devices by introducing precision-aware algorithms that maintain near plaintext accuracy. These advances validate that lightweight HE can be deployed at the network edge, paving the way for practical, edge-assisted encrypted learning as envisioned in EAH-FL.

Another thread of work focuses on INC for FL efficiency. Su et al. [11] proposed FediAC, a voting-based consensus model compression algorithm that reduces switch memory usage by aligning model indices before aggregation. Their results demonstrated faster convergence and reduced communication cost, but they left model updates unencrypted, creating privacy risks. Xia et al. [12] introduced GAIN, a stateless in-network aggregation framework that leverages programmable switches to accelerate FL, achieving up to 4.11× throughput and 86.5% traffic reduction. However, GAIN’s privacy guarantees came from architectural statelessness rather than cryptographic protection, making it inadequate for sensitive healthcare applications. Ji et al. [18] approached the same problem by modeling in-network aggregation and flow routing as an optimization problem. Their online scheduling framework minimized communication cost and delay, but like FediAC and GAIN, their solution assumed plaintext aggregation, overlooking privacy requirements. Zang et al. [13] extended INC concepts to IoT traffic analysis with FLIP4, combining lightweight models in network devices for fast, distributed learning. Although FLIP4 reduced communication costs and supported rapid incident response, it did not incorporate HE or encrypted aggregation, and it was designed for traffic analysis rather than healthcare. The need to merge these system-level optimizations with privacy guarantees has been emphasized in broader taxonomies of FL architectures by Caruccio et al. [14] and Albshaier et al. [15], both of whom identified edge-assisted and hybrid designs as key enablers for scalable, privacy-aware FL.

Surveys provide additional perspective on the field. Aziz et al. [16] offered a comprehensive review of HE and DP techniques for secure FL, highlighting their complementary roles in balancing privacy, convergence, and computation. Similarly, Albshaier et al. [15] systematically analyzed FL applications for cloud and edge security, revealing a 25% reduction in privacy risks but persistent communication bottlenecks. Xie et al. [19] reviewed efficiency optimization strategies for HE in FL, identifying batching, packing, and selective encryption as key techniques to reduce overhead. They concluded that HE is promising but impractical without system-level optimizations. Gu et al. [20] provided a comprehensive review of privacy-enhancing methods for healthcare FL, categorizing approaches such as HE, differential privacy, and secure aggregation. Their review underscored the lack of integrated frameworks that jointly address strong privacy and communication efficiency. Lessage et al. [21] contributed empirical evidence by applying FHE to FL for mammogram analysis, confirming that FHE preserved confidentiality but also revealed major memory and runtime limitations. Jin et al. [22] advanced the discussion with FedML-HE, which selectively encrypts sensitive parameters, reducing overhead by up to 40× for large models while maintaining resilience against inference attacks. Their results highlight selective encryption as a promising compromise between security and efficiency. A comparative summary of the discussed approaches is presented in Table 1, which contrasts their handling of privacy (HE), INC, latency, and scalability. As shown, existing systems typically address either privacy or efficiency in isolation, leading to an unresolved trade-off between cryptographic protection and real-time responsiveness.

Despite these contributions, healthcare-specific challenges remain. Wearable devices often collect highly heterogeneous and sometimes class-imbalanced data. One common scenario is the generation of one-class datasets, such as only normal or only abnormal samples, which destabilize global model training. Lee et al. [3] showed that FedAvg is relatively robust across diverse settings, but Yang et al. [2] demonstrated that additional mechanisms are still required for handling extreme heterogeneity in medical imaging. This confirms that healthcare IoT FL must simultaneously account for data imbalance, device constraints, and cryptographic overhead. The literature indicates that while progress has been made in both privacy and efficiency, there remains a clear gap in unifying the two. Collectively, these studies affirm that while significant progress has been made in algorithmic privacy and network optimization, a unified framework capable of delivering both end-to-end encryption and edge-level efficiency remains underexplored. To address this, we propose EAH-FL, which integrates edge-assisted HE with in-network aggregation. Unlike prior INC-based systems, EAH-FL ensures that all model updates remain encrypted throughout the network, eliminating the risks of plaintext aggregation. Unlike HE-only systems, it reduces prohibitive latency and bandwidth overhead through selective encryption and network-side aggregation. By offloading heavy cryptographic tasks to trusted edge devices, EAH-FL makes homomorphic FL feasible for resource-constrained wearables and demonstrates that secure, low-latency FL is achievable in real-time healthcare monitoring.

## 3. System Model

This section provides a formal description of the proposed secure, intelligent multi-stage pipeline. We define the network setup with all participating entities, state the underlying system-level assumptions (security, communication, resource, and trust), introduce the mathematical model (variables, equations, and constraints) that will be used later in the algorithmic implementation. This framework establishes the foundation for the algorithms and analyses in subsequent sections.

**Figure 1:**
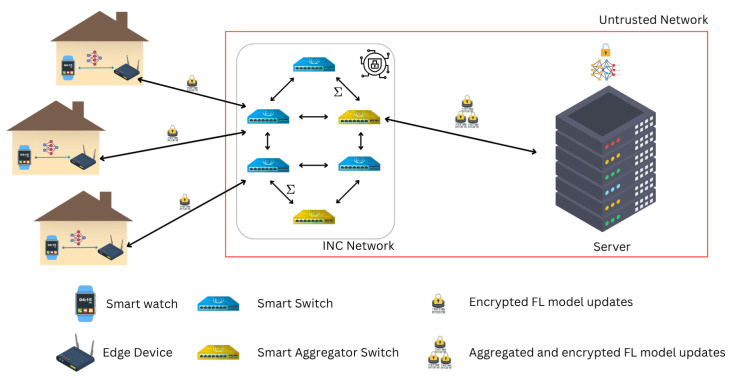
Proposed encrypted FL system with in-network aggregation.

Figure 1 illustrates the proposed encrypted federated learning (FL) system with in-network aggregation, mapping directly onto the four-tier architecture described above. On the **left**, the client tier (C) is represented by smartwatches, which *sense physiological data* such as heart rate, oxygen saturation, and activity levels. These clients transmit their raw measurements to the associated **edge nodes** (E), shown as home routers. Each edge node performs fully homomorphic encryption (FHE) on the received data, ensuring that all information leaves the local domain in encrypted form.

The encrypted updates are then forwarded into the **INC layer** (I), depicted as a network of interconnected smart switches and aggregator nodes. Unlike conventional forwarding, this layer performs two key roles: (1) *dynamic routing*, guided by a GNN-assisted policy that adapts paths based on network conditions, and (2) *in-network aggregation*, where encrypted updates from multiple edges are combined securely within the network fabric.Finally, on the **right**, the aggregated ciphertexts reach the **cloud server** (H), which updates the global FL model without decrypting individual client updates. The cloud performs encrypted aggregation across all participants, producing an updated global model. This updated model remains encrypted and is transmitted back downstream through the INC and edge nodes, eventually reaching the clients for local use. In the diagram, the encryption of updates is represented with *lock symbols*, highlighting that sensitive information is always protected. Multiple locks indicate contributions from different clients being aggregated securely. The arrows capture this end-to-end pipeline, showing the progression from data sensing at the client to encrypted global model updates, while emphasizing the points where security (FHE encryption) and intelligence (GNN-driven INC routing and aggregation) intervene in the workflow.

While Figure 1 provides a high-level view of the data flow for readability, the detailed technical realization is captured below in the following sections.

### 3.1. Network Setup and Entities

We consider a hierarchical, multi-layer edge computing architecture that integrates clients, edge nodes, INC, and cloud servers, each with distinct roles and responsibilities. Such multi-tier designs align with the fog computing paradigm, where an intermediate “fog” layer exists between the edge and the cloud in larger deployments. Formally, let C, E, I, and H denote the sets of clients, edge nodes, INC nodes, and cloud servers (respectively). For example, $C=c_{1},\ldots ,c_{N}$ is the set of *N* client devices, and similarly $E=e_{1},\ldots ,e_{M}$, $I=i_{1},\ldots ,i_{K}$, and $H=h_{1},\ldots ,h_{L}$ (often L=1 if a single cloud service is considered). We define a mapping f:C→E that associates each client $c_{i}$ with an edge node $e_{f(i)}$ responsible for that client (for instance, f(i)=j if client $c_{i}$ connects to edge $e_{j}$). Typically, this mapping reflects network proximity or pre-established pairing (e.g., a client’s personal or local gateway). Similarly, edge nodes are connected through the network to the cloud, possibly via multiple INC nodes. We may represent the network beyond the edge as a graph G=(V,E) where V=E∪I∪H and *E* are communication links; each edge node $e_{j}$ has one or more routing paths through INC nodes to reach the cloud h∈H. For simplicity, we often consider a representative path $P_{j}=\left[e_{j}\to i_{a}\to \ldots \to i_{b}\to h\right]$ for each edge $e_{j}$, where $i_{a},\ldots ,i_{b}\in I$ are the intermediate nodes on that route, terminating at the cloud *h*.

**Clients (C)**—The client layer consists of end devices, in our case a smartwatch, that generate data and service requests at the network edge. Clients are resource-constrained in computation, memory, and energy. They may perform minimal local processing (e.g., sampling or simple preprocessing) but offload heavy tasks to the edge node. In our pipeline, a client mainly collects raw data (heart rate, oxygen saturation, blood pressure, activity) and securely transmits it to its edge node. Clients operate at the first tier, typically connecting via Bluetooth, Wi-Fi, or cellular. A generic client is denoted as $c_{i}\in C$, with $D_{i}$ representing the data size (or task) generated. Due to limited capabilities, clients cannot handle intensive tasks (e.g., large-scale analytics or strong encryption) and thus rely on the edge node.

**Edge Nodes (E)**—Edge nodes are intermediate servers or gateways close to clients (e.g., home routers). Each client $c_{i}$ is paired with an edge node $e_{f(i)}$ that serves as its immediate processor and gateway. They bridge resource-limited clients and the core network by handling offloaded tasks such as data aggregation, format conversion, and cryptographic operations. Edge nodes also manage communication (e.g., packet encapsulation) and act as the first hop for data leaving the local domain. We denote $C_{e}$ as the compute capacity of edge node *e* (CPU cycles/s) and $B_{c_{i},e}$ as the bandwidth of the client–edge link. Practically, edge nodes (micro-servers or routers) have more resources than clients but less than the cloud. Processing at the edge reduces latency and network load, as only processed or compressed data may be forwarded. Clients typically trust their edge node, forming a local trust domain.

INC Layer (I)—INC nodes are network devices (e.g., smart routers, switches) with compute and storage capabilities, enabling in-network processing beyond simple forwarding. In our pipeline, they can aggregate results from edges, filter or fuse streams, and execute lightweight functions to reduce data volume before reaching the cloud. They also support dynamic routing and load balancing based on network conditions or policies. The INC layer (third tier) thus acts as a fog layer, offloading some cloud tasks and improving responsiveness by leveraging backbone resources. We denote the capacity of an INC node $i_{k}\in I$ as $C_{i_{k}}$, and bandwidths on edge–INC and INC–cloud links as $B_{e,i_{k}}$ and $B_{i_{k},h}$, respectively. While multiple INC nodes may exist along a path, we often treat the INC fabric as a single intermediate layer in analyses of delay or security. Operated by providers or third parties, INC nodes are modeled as semi-trusted (“semi-honest”) in our security framework. They remain central to the pipeline’s intelligence, enabling adaptive strategies (including AI-based controllers) for routing and aggregation.

**Cloud (H)**—The cloud layer (fourth tier) consists of centralized high-performance servers with virtually unbounded computation and storage. It executes global or compute-intensive tasks that exceed the edge or INC layers, such as aggregating results, running large-scale analytics, and finalizing model training. In our pipeline, the cloud acts as the global aggregator and back-end processor, collecting encrypted updates from distributed clients, combining or analyzing them, and producing final outputs or updated models. Results (e.g., inferences, decisions, or refined models) may then be sent back to edges and clients. We model the cloud as honest-but-curious: it follows the protocol but is not trusted with raw data, which arrives encrypted or sanitized. The cloud’s compute capacity is denoted $C_{h}$ (treated as unbounded), and its link bandwidth from INC or edge nodes as $B_{*,h}$. In short, the cloud provides centralized power and storage at the cost of higher latency and weaker trust.

These four entities form a pipeline: a client’s data or request flows upward through edge and INC nodes to the cloud, undergoing processing at each stage, and results flow back down to the client. By organizing the system into these layers, we achieve low-latency local processing at the edge, intelligent mid-layer optimization in the network, and heavy-duty processing plus global coordination at the cloud. Next, we outline the key assumptions about the environment, security, and trust in which this multi-stage pipeline operates.

### 3.2. Underlying Assumptions

Designing a secure, distributed pipeline requires several system-level assumptions to be established. We explicitly state the assumptions regarding the network environment, security model, resource limitations, trust relationships, and threat model. These assumptions are chosen to be realistic and to ensure a safe foundation for our system (they are not overly idealized), while avoiding any speculative or hypothetical results. The following are the principal assumptions underpinning our architecture:

1. **Communication Model:** We assume that clients connect to edge nodes over wireless links (e.g., a wearable device to a smartphone or home gateway via Bluetooth or Wifi). The edge nodes and INCs are interconnected through the broader network (which could include the internet or a private WAN). Standard networking protocols ensure basic data delivery between layers. However, network communication is not perfectly reliable—latency and packet loss can occur. We assume a packet-switched network where messages may be delayed, dropped, or reordered by adversarial conditions. Despite this, mechanisms like acknowledgments and retransmissions (or higher-level protocols) can provide eventual reliability. Time is divided into discrete intervals or rounds when analyzing performance (especially if considering synchronized operations like training rounds), but the system can also handle asynchronous requests. We also assume each link has a finite bandwidth $B_{x,y}$ and propagation delay; thus large messages incur transmission delays as introduced in the mathematical model later in this section.
2. **Resource Constraints:** Each tier has limited resources relative to its workload. Client devices have severe constraints on battery, CPU, memory, and thus cannot perform intensive computations or continuous heavy communication. This drives the need to offload tasks to the edge. Edge nodes have more computational power and energy (mains-powered), but they are still limited (e.g., a microserver with fixed CPU/GPU capacity that must be shared among multiple clients). INC nodes likewise have finite processing and storage—while they might be powerful network devices, they are not full data-center servers and typically must forward many flows, so any INC must be lightweight. The cloud has abundant resources but not infinite; in practice, we assume the cloud can scale to handle the aggregated workload of all clients, albeit with some upper bound or cost. We assume that no single edge or INC can handle an unbounded number of clients simultaneously without performance degradation; therefore, tasks may need to be distributed or queued if capacity is exceeded. In formulating our model, we will impose capacity constraints (e.g., an edge node $e_{j}$ cannot exceed $C_{e_{j}}$ CPU cycles per second across all tasks, and similarly for INC nodes). We also assume that storage at each layer is sufficient for buffering and intermediate data, but edge/INC storage is not large enough to hold full global datasets or models—such large-scale data resides in the cloud. This multi-tier resource distribution reflects common practice in edge computing, where moving computation closer to data sources improves latency but each step closer to the edge has smaller capacity.
3. **Security and Trust Boundaries:** We divide the system into trust domains. The client and its edge node form a fully trusted domain. Beyond this, the network—including INC nodes and links—is untrusted or semi-trusted. INC nodes are semi-trusted: they execute assigned tasks (e.g., aggregation, routing) correctly but may be curious or compromised. They do not intentionally corrupt computations but cannot be trusted with confidential data. The cloud is modeled as honest-but-curious: it follows protocols correctly but may analyze data to infer private information, so raw client data must not be exposed.Communication channels are fully untrusted, subject to eavesdropping, tampering, and replay attacks. To ensure confidentiality and integrity, all data leaving the edge is encrypted end-to-end with CKKS (FHE), and entities are authenticated (via certificates or pre-shared keys). Within the client–edge domain, plaintext handling is permitted under the assumption of physical security. In summary: the client–edge pair is trusted, INC is semi-trusted, the cloud is honest-but-curious, and network channels are untrusted. Threats include passive adversaries, compromised INC nodes, and packet injection or alteration. Standard cryptographic primitives are assumed secure. Denial-of-service attacks are possible but out of scope; we focus on confidentiality, integrity, and correct protocol execution.
4. **Task and Application Model:** We assume that the workload can be broken into tasks or data units that flow through the pipeline. Each client may generate tasks (or data updates) periodically or in response to events. We make the simplifying assumption that each task from a client is independent in terms of scheduling (though they could be part of a larger application workflow). The pipeline supports a variety of applications, for example, real-time sensor data aggregation, federated machine learning updates, augmented reality offloading, etc.— but in all cases, the pattern is that raw data originates at the client and the final processing is needed at the cloud or at least beyond the edge. The communication pattern is often uplink-heavy: clients send data up the chain and eventually receive some result or acknowledgment back. We assume the volume of data returned from the cloud (downlink) is relatively small (an updated model) compared to the volume of data uploaded, which is common in sensor analytics and learning scenarios. This justifies our focus on the upstream data pipeline for performance modeling. If a specific application requires heavy downlink data (e.g., content delivery), a similar analysis can be applied in reverse. Another assumption is that tasks might have real-time requirements—for instance, each task *i* may have a deadline or latency requirement $\Delta_{i}$ that the end-to-end processing must meet (especially in mission-critical contexts). We assume such requirements are known and form part of the constraints in our system design (e.g., the pipeline and algorithms should aim to ensure $T_{i}\leq \Delta_{i}$ for each task’s completion time $T_{i}$ as defined later).
5. **Consistency and Fault Tolerance:** We assume a consistent view of the system in terms of configuration, e.g., the mapping $f\left(c_{i}\right)=e_{j}$ does not change frequently (a client is generally served by the same edge node, unless it moves and hands off to a new edge, which we assume happens relatively infrequently). INC routing paths are assumed to be known or can be discovered by the network controllers. If a node fails (edge, INC, or cloud), we assume there are failover mechanisms (out of scope for our model) that eventually reroute tasks to an alternate node of the same tier. Our focus is on steady-state operation under normal conditions and under security threats, rather than on recovery from node crashes. However, we do assume that the system is distributed—no single point of failure should halt the entire pipeline. For example, if one INC node on a path is down, another INC or an alternate route can be used (this will be orchestrated by the network’s intelligent Routing Algorithm discussed in Section 5).

With these assumptions in place, our pipeline is designed to function both securely and efficiently. We now turn to establishing the foundation for the encryption used in our model.

### 3.3. Homomorphic Encryption Setup

We use FHE with the CKKS scheme as implemented in TenSEAL to enable approximate arithmetic directly over encrypted floating-point values. This choice allows model parameters and health data streams to remain encrypted end-to-end while still supporting aggregation and statistical operations required for real-time analysis. Furthermore, to ensure strict reproducibility, a global seed s = 42 is applied consistently across model initialization, data partitioning, and training routines. This guarantees deterministic experimental outcomes.

#### 3.3.1. Cryptographic Context

The encryption context is defined by the tuple

(1) P=(N,Δ,Q)

where

- **Polynomial modulus degree: N=8192**
  – Governs ciphertext size and security strength.
  – Provides 128-bit security against RLWE attacks.
  – Large enough to encode multiple model parameters in a single ciphertext (via SIMD packing), which is essential for vectorized aggregation in INC.
  – Smaller values (N=4096) reduce latency but fall short of the security level required; larger values (N=16,384) increase computational overhead beyond real-time feasibility.

- **Scaling factor: $\Delta =2^{20}$**
  – Controls fixed-point precision of encrypted computations.
  – A scaling factor of $2^{20}$ yields 6 decimal digits of accuracy, sufficient for model parameter aggregation where minor floating-point deviations do not affect convergence.
  – Higher scaling factors increase precision but lead to faster noise growth, limiting computation depth.

- **Coefficient modulus chain: Q=[40,40,40,40]**
  – Defines the sequence of modulus sizes for rescaling.
  – This chain supports multiple levels of additions and multiplications before noise exhaustion, while keeping ciphertext size manageable.
  – The symmetric structure [40,40,40,40] provides balanced noise distribution across rescaling steps, enabling secure aggregation of parameters through several INC hops.

Formally, the plaintext encoding is defined as:

(2) $$C^{N/2}\;\overset{\;\mathrm{Encode}\;}{\to }\;R_{N}=Z\left[X\right]/\left(X^{N}+1\right),$$

where Z[X] denotes the ring of polynomials in *X* with integer coefficients, and *N* is the polynomial modulus degree. The quotient $\left(X^{N}+1\right)$ defines the cyclotomic polynomial used to bound polynomial degrees.

#### 3.3.2. Secure Dataflow in the Pipeline

All sensor data is encrypted once at the router using the CKKS context P(1). From this point forward, ciphertexts are never decrypted until they are relayed back to the client device Within the INC fabric, smart switches and intermediate nodes operate only over ciphertexts, performing homomorphic additions and multiplications such as

(3) $$\mathrm{Enc}\left(\theta \right)\;\mapsto \;\sum_{i=1}^{m}\mathrm{Enc}\left(\theta_{i}\right),$$

where $\mathrm{Enc}(\theta_{i})$ are encrypted parameter vectors aggregated hop-by-hop. Because CKKS supports (Single Instruction Multiple Data) SIMD style packing, multiple parameters can be aggregated in parallel, enabling efficient streaming across IoT topologies.

Next, we formalize the mathematical model of the pipeline, introducing variables and equations that characterize communication delays, processing times, resource constraints, and the objective functions that drive the intelligent orchestration of this multi-stage system.

### 3.4. Mathematical Model

Before presenting the formal mathematical model, it is important to highlight that our system integrates two distinct, yet complementary, computational domains. The first domain is the *federated learning (FL) framework*, which governs how model updates are generated on end devices and securely aggregated through the pipeline up to the cloud. The second domain is the *graph neural network (GNN)-based routing framework*, which dynamically determines optimal data forwarding and aggregation strategies within the INCs. This division allows us to gradually introduce the notation and variables specific to each domain while ensuring a coherent integration of both perspectives in the overall system model.

#### 3.4.1. Federated Learning Framework

We consider a hierarchical federated learning pipeline where updates flow from clients *C* through edges *E* and INC nodes *I* to the cloud *H*.

At the client layer, each $c_{i}\in C$ holds a private dataset

(4) $$D_{i}=\left\{\left(x_{ij},y_{ij}\right)|j=1,\ldots ,n_{i}\right\},$$

with $x_{ij}\in R^{d}$ features and $y_{ij}\in \left\{0,1\right\}$ labels. The local model is parameterized by θ=(w,b), where *w* is the weights and *b* is the bias. In each round *t* the client produces parameters $\theta_{i}^{t}$ with a paired edge $e_{f(i)}\in E$. The edge initializes the CKKS context P(1), generates keys (pk−publickey,sk−secretkey), and encrypts the local parameters:

(5) $$\left[\left[\theta_{i}^{t}\right]\right]=\mathrm{Enc}\left(\theta_{i}^{t};pk,P\right).$$

From this step onward, all communication is encrypted.

The encrypted updates traverse paths $P_{j}=\left[e_{j}\to i_{a}\to \ldots \to i_{b}\to h\right]$ through the INC layer (the path is decided by a dynamic machine learning algorithm that we discuss in the next section), where $i_{a},\ldots ,i_{b}\in I$ and h∈H. INC nodes perform homomorphic aggregation without decryption:

(6) $$\left[\left[\theta^{t}\right]\right]=\sum_{i\in R_{t}}\frac{n_{i}}{N_{t}}\cdot \left[\left[\theta_{i}^{t}\right]\right],$$

where $R_{t}\subseteq C$ are the participating clients in round *t* and $N_{t}=\sum_{i\in R_{t}}n_{i}$.

At the cloud server h∈H, the aggregated ciphertext updates the global FL model while it is encrypted:

(7) $$\left[\left[\theta^{t+1}\right]\right]=\mathrm{UpdateEncrypted}\left(\left[\left[\theta^{t}\right]\right]\right).$$

The newly trained global model is then returned to the edge $e_{f(i)}$, which decrypts with sk:

(8) $$\theta^{t+1}=\mathrm{Dec}\left(\left[\left[\theta^{t+1}\right]\right];sk\right),$$

and synchronizes plaintext global parameters $\left(W^{t},b^{t}\right)$ back to clients.

Thus, the mathematical flow of federated learning can be summarized as:

(9) $$\theta_{i}^{t}\;\overset{\;e_{f(i)}\;}{\to }\;\left[\left[\theta_{i}^{t}\right]\right]\;\overset{\;I\;}{\to }\;\left[\left[\theta^{t}\right]\right]\;\overset{\;h\;}{\to }\;\left[\left[\theta^{t+1}\right]\right]\;\overset{\;e_{f(i)}\;}{\to }\;\theta^{t+1}.$$

This formulation establishes the core mathematical model and end-to-end data flow for our federated learning loop under HE. In Section 4, we extend this baseline into our proposed EAH-FL framework, where the same primitives are embedded into a structured algorithm. There, we formalize the update procedure, introduce edge-assisted coordination mechanisms, and analyze how these refinements improve efficiency, scalability, and security while maintaining full encryption throughout the pipeline.

#### 3.4.2. Graph-Based Representation for Adaptive Routing

While the FL framework ensures end-to-end confidentiality through HE, the underlying communication layer must also operate efficiently under limited resources. FL with HE increases computational and transmission overhead, making intelligent routing essential especially for INC nodes and switches that must dynamically forward encrypted packets without excessive delay or packet loss. Routing in such an environment can be naturally formulated as a graph learning problem. Each node’s forwarding decision depends not only on its own state but also on the states of its neighboring nodes. This dependency structure aligns with the message-passing mechanism of GNNs, which aggregate neighborhood information to learn context-aware node representations useful for routing.

To formalize this, we model the INC fabric as a directed graph

(10) G=(V,E),|V|=N,

where V is the set of network nodes and E the set of directed communication links. Each node u∈V is associated with a feature vector

(11) $$z_{u}=\left[E_{u},L_{u},T_{u},B_{u}\right],$$

capturing residual energy $\left(E_{u}\right)$, connectivity degree $\left(L_{u}\right)$, trust score $\left(T_{u}\right)$, and buffer occupancy $\left(B_{u}\right)$. Collectively, these features form the matrix

(12) $$Z\in R^{N\times d},$$

where *d* denotes the dimensionality of node features.

A generic *K*-layer Graph Neural Network then maps the pair (Z,A) to node embeddings through iterative message passing. Each layer updates node representations as

(13) $$H^{\left(k+1\right)}=\sigma \left(AH^{\left(k\right)}W^{\left(k\right)}\right),$$

where A is the normalized adjacency matrix of G, $W^{\left(k\right)}$ denotes the trainable weight matrix of layer *k*, and σ(·) is a nonlinear activation such as ReLU. This operation aggregates information from each node’s neighbors and transforms it into a higher-level representation capturing both local and topological context. After *K* iterations, the final embeddings are expressed as

(14) $$H^{\left(K\right)}={\mathrm{GNN}}_{K}\left(Z,A\right).$$

The resulting embeddings $H^{\left(K\right)}$ encapsulate each node’s structural and functional context and are subsequently used to compute adaptive routing scores or next-hop probabilities. This formulation forms the foundation of the Adaptive Multi-Relational Routing Graph Neural Network (AMR-GNN) introduced in Section 5.

The mathematical framework in Section 3 integrates federated learning, homomorphic encryption, and graph-based routing into a unified system model. It defines the operational roles and constraints of clients, edges, INC nodes, and the cloud, establishing the foundation for end-to-end secure and efficient communication. Section 4 expands this model into the Edge-Assisted Homomorphic Federated Learning (EAH-FL) framework, while Section 5 extends it further by embedding the GNN-based routing mechanism, demonstrating through experiments that the proposed system achieves both privacy preservation and efficient in-network operation.

## 4. Edge-Assisted Homomorphic Federated Learning

Building directly on the system model and cryptographic foundations introduced in Section 3, we now formalize the federated learning protocol that enables privacy-preserving model training over wearable health data. The focus of this section is to connect the architectural assumptions of Section 3 trusted edge devices, programmable in-network aggregation, and CKKS-based encryption to the end-to-end training pipeline and its practical realization.

In particular, we detail three key aspects of the proposed EAH-FL design:

1. The complete encrypted training pipeline that operates across clients, edges, and the cloud (Section 4.1),
2. Stabilization strategies for one-class clients to ensure convergence in heterogeneous data settings (Section 4.2)

Throughout this section, we retain the notation of Section 3: a federation of *n* clients indexed by *i*, each with dataset $D_{i}$ of size $n_{i}$, and global parameters $\theta^{t}=\left(W^{t},b^{t}\right)$ at round *t*. Ciphertexts are denoted by 〚·〛 under the CKKS scheme with public/private keys (pk,sk) generated at the edge. The cloud remains honest-but-curious and never holds sk, ensuring that aggregation and global updates are performed exclusively in the encrypted domain.

### 4.1. Complete Training Pipeline

Algorithm 1 presents the full training workflow of the proposed EAH-FL framework. The system operates over *T* federated communication rounds, indexed by t=1,…,T. At the start, a global model is initialized at the edge using logistic regression parameters $\left(W_{0},b_{0}\right)$, and the CKKS encryption context is configured (e.g., polynomial modulus degree, scaling factor). The edge device also generates a public/private key pair (pk,sk) for encryption/decryption. In each round *t*, the following steps are executed, corresponding to the loop in Algorithm 1:

1. **Client-side local training:** Each client *i* loads and preprocesses its private dataset $D_{i}$. The clients are trained on 55 engineered features using logistic regression. If the client exhibits one-class behavior (only positive or only negative samples), one-class handling strategies are applied (see line 6 of Algorithm 1). Clients then perform local training to obtain $\left(w_{i}^{t},b_{i}^{t}\right)$, i.e.,
  (15) $$\theta_{i}^{t}=\left[w_{i}^{t},b_{i}^{t}\right].$$
2. **Edge-side encryption:** The plaintext model parameters $\theta_{i}^{t}$ in (15) are sent to the edge device, which encrypts them under the public key pk and context P(1) to produce
  (16) $$\left[\;\left[\theta_{i}^{t}\right]\;\right]=\mathrm{Enc}\left(\theta_{i}^{t};pk;P\right).$$
  this is similar to Equation (5) we discussed in the mathematical model
3. **INC-side encrypted aggregation:** The encrypted updates from all clients are forwarded to an INC node (see line 18 of Algorithm 1). The INC node performs homomorphic aggregation to produce
  (17) $$\left[\;\left[\theta^{t}\right]\;\right]=\sum_{i\in R_{t}}\left(\left[\;\left[\theta_{i}^{t}\right]\;\right]\cdot \frac{n_{i}}{N_{\mathrm{tot}}}\right),$$
  where $N_{\mathrm{tot}}=\sum_{i\in R_{t}}n_{i}$.
4. **Cloud-side encrypted global update:** The aggregated ciphertext $\left[\;\left[\theta^{t}\right]\;\right]$ from (17) is relayed to the cloud, which completes the global model update in the encrypted domain to form
  (18) $$\left[\;\left[W^{t},b^{t}\right]\;\right].$$
5. **Edge-side decryption and client sync:** Each edge decrypts the encrypted global model (18) using its private key sk, recovering the plaintext parameters
  (19) $$\left(W^{t},b^{t}\right)=\mathrm{Dec}\left(\left[\;\left[W^{t},b^{t}\right]\;\right];sk\right),$$
  which are then synchronized to clients for the next round (see line 26 of Algorithm 1).

**Algorithm 1: **EAH-FL

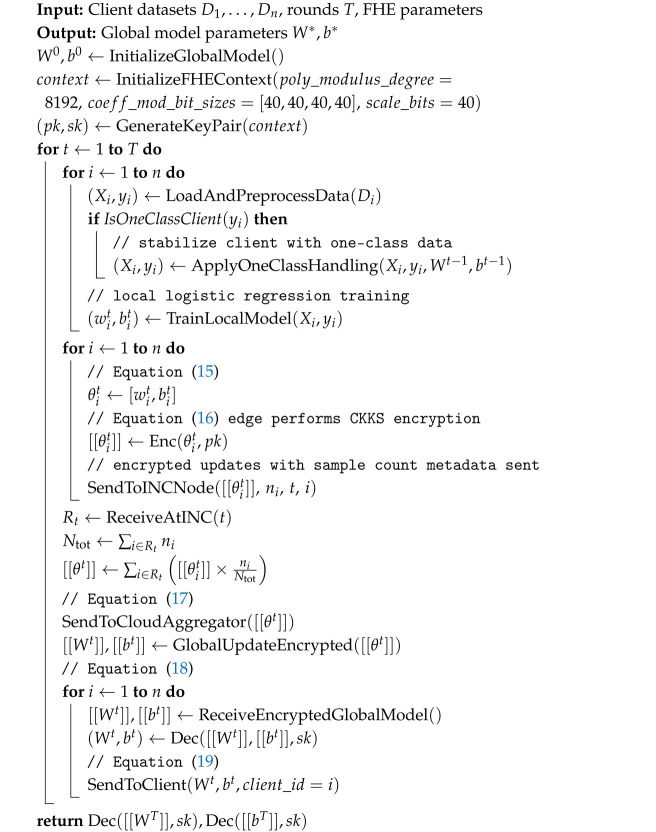



    Equations (15)–(19) correspond directly to the steps of Algorithm 1. This design ensures that client updates remain confidential throughout the pipeline. At no point does the cloud observe raw model weights or gradients. The trusted edge offloads cryptographic computation from resource-constrained devices, while the INC layer reduces WAN traffic via ciphertext fan-in, enabling practical and secure FL in healthcare IoT environments.

### 4.2. One-Class Client Handling

A key challenge in healthcare FL is clients with data from only one class (e.g., no positive cases). These clients can destabilize global model training if not properly handled. EAH-FL includes a dedicated mechanism for this scenario (Algorithm 2).

**Algorithm 2: **One-Class Client Handling (Combined Strategy)

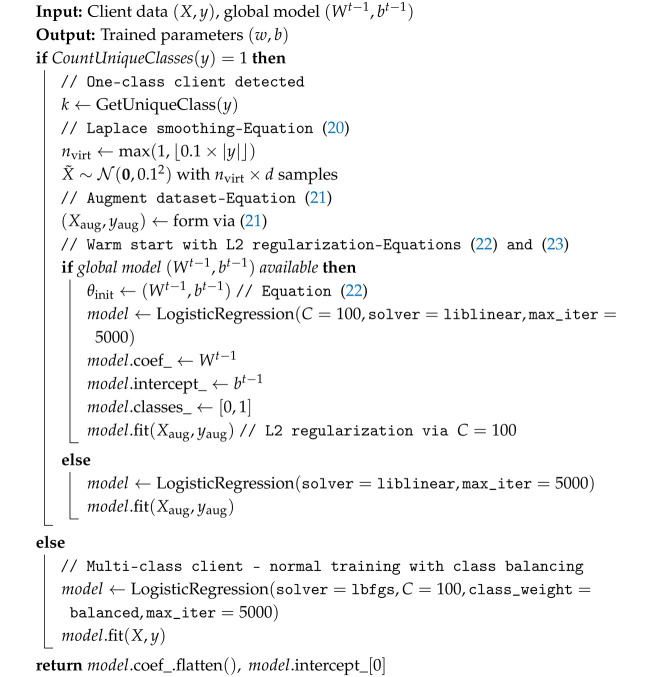



    **Problem Setup:** For client *i*, let the local dataset be

$$D_{i}={\left\{\left(x_{j},y_{j}\right)\right\}}_{j=1}^{n_{i}},\;y_{j}\in \left\{0,1\right\},$$

where $n_{i}$ is the number of samples. If all labels are equal to some class k∈{0,1} (i.e., $\left\{y_{j}:j=1,\ldots ,n_{i}\right\}=\left\{k\right\}$), we say client *i* has a “one-class problem”. We denote the missing class by 1−k.

**Laplace Smoothing:** To address the one-class issue, we generate synthetic samples for the missing class. - Let α>0 be a smoothing parameter (we use α=0.1 in our implementation). - The number of synthetic samples is

(20) $$n_{\mathrm{virt}}\;=\;\max\;\left(1,\;\lfloor \alpha \;n_{i}\rfloor \right).$$

- Let $\tilde{X}∼N\left(0,0.1^{2}\right)$ be $n_{\mathrm{virt}}\times d$ Gaussian random features for class 1−k. - The augmented dataset is

(21) $$X^{\mathrm{aug}}\;=\;\left[\begin{array}{c} X\\ \tilde{X} \end{array}\right],\;y^{\mathrm{aug}}\;=\;\left[\begin{array}{c} y\\ \left(1-k\right)1_{n_{\mathrm{virt}}} \end{array}\right],\;n_{i}^{\mathrm{aug}}\;=\;n_{i}+n_{\mathrm{virt}}.$$

**Warm-Start Initialization with L2 Regularization:** For one-class clients, we combine Laplace smoothing with warm-start initialization and L2 regularization:

(22) $$\theta_{\mathrm{init}}\;=\;\theta^{t-1}=\left(W^{t-1},b^{t-1}\right).$$

We train on the augmented dataset $\left(X^{\mathrm{aug}},y^{\mathrm{aug}}\right)$ with L2 regularization:

(23) $$L\left(\theta \right)=L_{\mathrm{data}}\left(\theta ;X^{\mathrm{aug}},y^{\mathrm{aug}}\right)+\lambda {∥\theta ∥}_{2}^{2},$$

where the regularization coefficient, denoted as λ, is set to 0.01. In logistic regression, the regularization strength is governed by the parameter *C*, calculated as C=1/λ, which results in a value of 100. This methodology enhances the stability of local updates by commencing from the parameters of the global model and incorporating L2 regularization throughout the training process. This approach effectively mitigates excessive divergence from the global parameters, while simultaneously allowing the model to leverage knowledge from the augmented data.

## 5. Adaptive Multi-Relational Routing Graph Neural Network for Smart Networks

After establishing the secure transmission layer through HE and refining local models within the federated learning loop, the next challenge lies in capturing the behavior of the underlying INC layer. Since the efficiency of the overall system depends not only on data confidentiality but also on how packets traverse and aggregate across the network, we now turn to the problem of routing. To address this, we introduce the AMR-GNN, which adapts decisions to evolving network states. We then complement this with a heuristic-imitation framework, where the GNN is trained to replicate the choices of a rule-based policy. Together, these two perspectives allow us to contrast adaptive learning with heuristic guidance, and provide a foundation for evaluating the trade-offs between stability, interpretability, and performance in our INC setting.

### 5.1. Dynamic Routing

Building on the graph-based routing model defined in Section 3.4.2, we now specialize the GNN architecture to the requirements of INC under homomorphic federated learning. While the generic formulation $H^{\left(K\right)}={\mathrm{GNN}}^{\left(K\right)}\left(Z,A\right)$ yields structural embeddings of the INC fabric, it does not capture the heterogeneous semantics of nodes and links in this setting. Routing decisions must jointly account for residual energy, encryption overhead, latency, and trust, while edges must reflect variable bandwidth and reliability.

For each node *u*, the hidden representation at layer *k* is updated as:

(24) $$h_{u}^{\left(k\right)}=\sigma \left(W^{\left(k\right)}\cdot \left[h_{u}^{\left(k-1\right)}\;∥\;\mathrm{AGG}\left\{h_{v}^{\left(k-1\right)}:v\in N\left(u\right)\right\}\right]\right),$$

with $h_{u}^{\left(0\right)}=z_{u}$, σ(·) an activation function, and AGG(·) an aggregation operator. After *K* layers, the enriched embeddings $H^{\left(K\right)}$ encode both structural and contextual information, tailored for routing.

The routing policy is parameterized as:

(25) $$\pi \left(a_{u}\;|\;h_{u}^{\left(K\right)}\right)=\mathrm{softmax}\left(W_{o}h_{u}^{\left(K\right)}\right),$$

where $\pi (a_{u}\;|\;h_{u}^{\left(K\right)})$ denotes the probability of forwarding from node *u* to a neighbor $a_{u}$.

Finally the objective is to balance delivery, latency, energy, and trust. We capture this trade-off through a composite reward:

(26) R=α·PDR−β·Delay−γ·Energy+δ·Trust,

with coefficients α,β,γ,δ reflecting system priorities. This reward guides both training and runtime evaluation of AMR-GNN policies.

Algorithm 3 operationalizes the mathematical formulations in Equations (10)–(14) and (24)–(26) within the live INC fabric. Starting from the graph representation G=(V,E) (Equation (10)), each node u∈V constructs its feature vector $z_{u}=\left[E_{u},L_{u},T_{u},B_{u}\right]$ (Equation (11)), capturing residual energy, connectivity degree, trust score, and buffer occupancy. These features are aggregated into the feature matrix $Z\in R^{N\times d}$ (Equation (12)), which is then propagated through a *K*-layer GNN to produce enriched embeddings $H^{\left(K\right)}={\mathrm{GNN}}_{K}\left(Z,A\right)$ (Equation (14)). During propagation, hidden representations $h_{u}^{\left(k\right)}$ are updated layer by layer according to Equation (24), integrating both structural and contextual information from neighbors. The resulting embeddings parameterize the routing policy $\pi \left(a_{u}|h_{u}^{\left(K\right)}\right)=\mathrm{softmax}\left(W_{0}h_{u}^{\left(K\right)}\right)$ (Equation (25)), which assigns forwarding probabilities over candidate neighbors. Instead of uniform neighbor selection, the algorithm ranks neighbors using logits from π and restricts forwarding to the top-*K* candidates, applying a loop penalty to previously visited nodes to avoid cycles. Final action selection is sampled from this top-*K* set with softmax temperature scaling. At each hop, encrypted updates are combined in-network using the homomorphic operator HE_SUM(·), enabling secure aggregation of ciphertexts before reaching the cloud. Routing is further guided by the composite reward R=α·PDR−β·Delay−γ·Energy+δ·Trust (Equation (26)), balancing delivery, latency, energy efficiency, and trust in real time.

### 5.2. Heuristic-Imitation Training

When training a GNN to make forwarding decisions, relying only on reinforcement signs can be slow and unstable, since the model must explore before it discovers effective strategies. To accelerate this process, we let the network first imitate a simple heuristic: a rule-based neighbor selection that encodes intuitive preferences such as avoiding congested nodes, exploiting aggregation opportunities, and moving closer to the server. By imitating these choices, the GNN quickly learns useful Patterns that form a strong starting point for further optimization. In order to gain deeper insight into the local training dynamics of the GNN, we now introduce the corresponding mathematical equations with explanatory notes.

The queue length at a node reflects the number of packets currently waiting to be processed. To avoid biasing high-degree nodes, we normalize it by the degree of the node:

(27) $$q_{n}\left(v\right)\;=\;\frac{\mathrm{queue}[v]}{\max\;\left(1,\;\sum_{j}\mathrm{adj}\left[v,j\right]\right)}$$

where queue[v] is the queue size at node *v* and $\sum_{j}\mathrm{adj}\left[v,j\right]$ is its degree.

The aggregation potential quantifies how well a node can combine traffic from multiple neighbors. It counts the number of active neighbors with non-empty queues:

(28) $$\mathrm{aggr}\left(v\right)\;=\;\sum_{u:\;\mathrm{adj}[v,u]=1}1\;\left[\;\mathrm{queue}[u]>0\;\right]$$

The dynamic feature vector summarizes key local conditions of a node—its utilization, normalized queue, and aggregation capability:

(29) $$\mathrm{dyn}\left(v\right)\;=\;\left[\;\mathrm{util}\left[v\right],\;q_{n}\left(v\right),\;\mathrm{aggr}\left(v\right)\;\right]$$

**Algorithm 3: **INC Round: Dynamic Routing and Homomorphic Aggregation

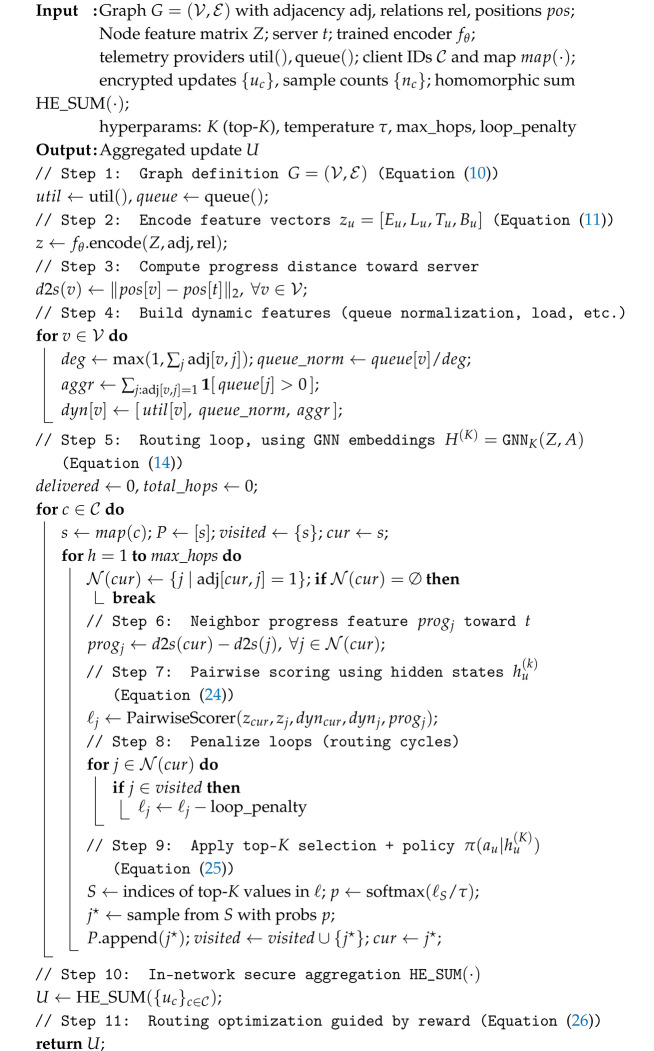



    The Euclidean distance to the server is used as a spatial progress measure, since nodes closer to the server are preferable for forwarding:

(30) $$d2s\left(v\right)\;=\;{∥\;\mathrm{pos}[v]-\mathrm{pos}[t]\;∥}_{2}$$

The relative progress of forwarding from node *i* to neighbor *j* is given by:

(31) $${\mathrm{prog}}_{ij}\;=\;d2s\left(i\right)-d2s\left(j\right)$$

Positive values indicate advancement toward the server, while negative values correspond to detours.

The heuristic score evaluates each neighbor based on weighted factors of utilization, queue state, aggregation potential, and progress:

(32) $$s_{ij}\;=\;-w_{u}\;{\mathrm{util}}_{j}\;-\;w_{q}\;q_{n}\left(j\right)\;+\;w_{a}\;\mathrm{aggr}\left(j\right)\;+\;w_{p}\;{\mathrm{prog}}_{ij},\;j\in N\left(i\right)$$

The heuristic label selects the neighbor with the maximum score, which acts as the imitation target for training:

(33) $$y_{i}\;=\;\left\{\begin{array}{cc} \arg\max_{j\in N(i)}s_{ij}, & \mathrm{if}\;i\neq t\;\mathrm{and}\;|N(i)|>0,\\ -1, & \mathrm{otherwise} \end{array}\right.$$

The model predicts logits for each edge by combining node embeddings, dynamic features, and progress information:

(34) $$\ell_{ij}\;=\;\mathrm{MLP}\;\left(\;[z_{i},\;z_{j},\;\mathrm{dyn}\left(i\right),\;\mathrm{dyn}\left(j\right),\;{\mathrm{prog}}_{ij}]\;\right),\;j\in N\left(i\right)$$

These logits are turned into probabilities over neighbors via the softmax function:

(35) $$p_{i}\;=\;\mathrm{softmax}\left(\ell_{i}\right)\;,\;\mathrm{where}\;{\left[\ell_{i}\right]}_{j}=\ell_{ij}$$

The cross-entropy imitation loss penalizes the model whenever it assigns low probability to the heuristic-chosen neighbor:

(36) $$L_{\mathrm{CE}}\;=\;-\sum_{i\in V_{\mathrm{valid}}}\log p_{i}\left[y_{i}\right]$$

To avoid degenerate predictions (e.g., always picking the same neighbor with near certainty), a variance regularizer encourages smoother probability distributions:

(37) $$L_{\mathrm{var}}\;=\;\sum_{\;i:\;|N(i)|>1}\mathrm{Var}\;\left(p_{i}\right)$$

Finally, the total training loss combines cross-entropy and variance terms, normalized over the number of contributing nodes:

(38) $$L\;=\;\frac{1}{m}\;L_{\mathrm{CE}}\;+\;\lambda \;\frac{1}{k}\;L_{\mathrm{var}}$$

where *m* is the number of valid nodes, *k* is the number with multiple neighbors, and λ balances the two objectives.

Algorithm 4 implements the offline heuristic-imitation training procedure for the INC-GNN, making use of both the newly introduced Equations (27)–(38) and the previously defined Formulations (11)–(14) and (24)–(26). First, node-level descriptors are reconstructed from graph snapshots: queue-normalized load $q_{n}\left(v\right)$ (27), aggregate neighbor activity aggr(v) (28), and the dynamic feature vector dyn(v) (29). Each node also computes its distance to the server d2s(v) (30) and relative progress ${\mathrm{prog}}_{ij}$ (31). These statistics feed into the heuristic scoring function $s_{ij}$ (32), from which pseudo-labels $y_{i}$ are assigned according to (33). Once labels are fixed, graph features are encoded into latent embeddings *z* via the GNN encoder using Equations (11)–(14), which follow the same propagation dynamics as the runtime model. For each valid node, neighbor logits $\ell_{ij}$ are produced through the pairwise MLP in (34), and normalized with the softmax rule (35). Training then minimizes the cross-entropy imitation loss $L_{\mathrm{CE}}$ (36), while a variance penalty $L_{\mathrm{var}}$ (37) ensures non-degenerate neighbor distributions. The overall composite objective is defined in (38) and optimized by Adam. In contrast to the runtime policy $\pi (a_{i}|h_{i}^{\left(K\right)})$ of Equation (25), which selects next-hop actions online, Algorithm 4 learns parameters θ offline by aligning model predictions with heuristic supervision. This training stage equips the INC-GNN with stable, generalizable routing behavior, which can then be deployed in real-time execution (Algorithm 3) under the performance-driven objective *R* in Equation (26).

The next section details the simulation environment, followed by a presentation and analysis of the results achieved using the proposed approach.

**Algorithm 4:** Heuristic-Imitation Training for INC-GNN

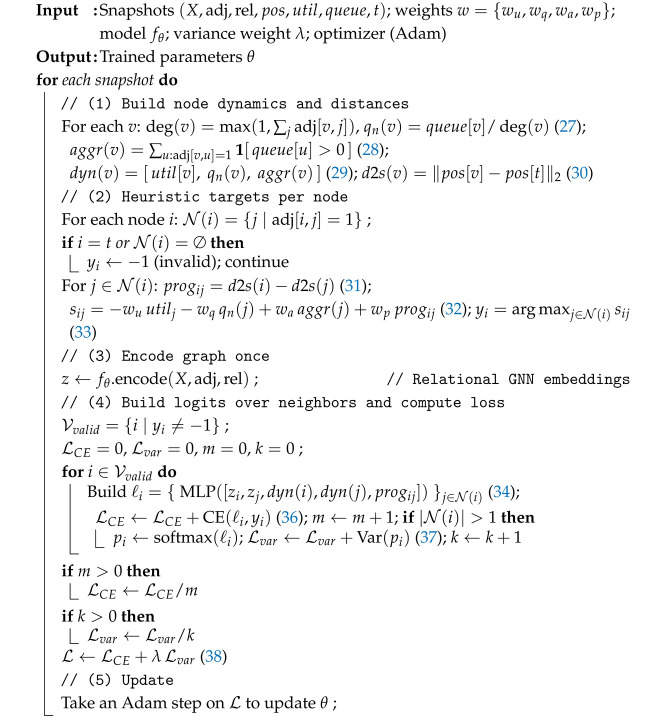



## 6. Performance Evaluation

### 6.1. Simulation Setup

We simulate cross-device federated binary classification on a large-scale *Kaggle FitLife Health and Fitness Tracking* dataset  [24] (≈600k records). Labels are derived by median-thresholding the continuous fitness_level attribute into a binary health status ∈{0,1}. The feature space consists of 55 engineered predictors: 13 basic physiological and behavioral variables, 24 derived ratios and composite features, 10 polynomial and square-root transformations, and 8 discretized categorical bins. To ensure consistent normalization across participants, a StandardScaler is fit on the union of client datasets. Each simulated client corresponds to one participant’s records, with a minimum requirement of 200 samples; clients below this threshold are excluded but logged. The number of simulated clients is configurable, with experiments conducted for |Clients|∈{10,20,30,40,50,60}. Federated learning rounds are similarly varied, with R∈{10,20,30,40,50,60}, in increments of 10, while maintaining identical partitions across runs for comparability. Local models are implemented as logistic regression classifiers (scikit-learn).

One-class client shards are handled using an combined strategy Laplace-style virtual minority sampling, warm-start initialization from the current global model, L2 regularization to maintain proximity to the global parameters, and a sample-weight floor to ensure minimum influence in the aggregation process. This ensures that clients with anomalous or imbalanced datasets are retained rather than excluded, thereby mitigating bias. On the server side, encrypted client updates are aggregated homomorphically using sample-count–weighted federated averaging, where each update is represented as the concatenation of weights and bias terms. The aggregation is performed directly on ciphertexts, and the global model is updated without decryption. For evaluation, the ciphertext is decrypted only after aggregation. Two experimental pipelines are executed: (1) a plaintext baseline and (2) a full end-to-end HE variant. The HE configuration employs a polynomial modulus degree of 8192 and a scale of 40 bits. Metrics evaluated over *R* rounds include Accuracy, F1-score, Precision, Recall, Area Under the Curve (AUC), total round time, and cryptographic overhead. Additionally, we log per-round encryption, aggregation, and total execution times to quantify scalability and performance impacts of HE. All simulations were conducted on a MacBook Pro (M4 chip, 24 GB RAM).

#### Ethical and Licensing Considerations

We are using public *FitLife Health and Fitness Tracking* dataset [24] for all the simulations. The dataset is hosted on Kaggle (https://www.kaggle.com/datasets/jijagallery/fitlife-health-and-fitness-tracking-dataset (accessed on 30 September 2025)) and released under the **CC0: Public Domain** license, permitting unrestricted use and redistribution for research and educational purposes. All records are fully anonymized and synthetic, containing no personally identifiable or protected health information. All processing complied with the dataset’s CC0 license terms and Kaggle’s data usage policies.

### 6.2. Evaluation Metrics

We adopt a range of widely used metrics from FL, INC, and wireless sensor network (WSN) literature to evaluate model utility and system performance comprehensively.

**Model performance** is first assessed using **accuracy**, the fraction of correctly classified samples, reported under both plaintext and encrypted FL settings for varying client sizes (10–60). To provide deeper insight into classification quality especially relevant for imbalanced IoT and healthcare datasets—we also report **precision**, **recall**, and the **F1-score**. Precision is the proportion of true positives among predicted positives, recall is the proportion of true positives among all actual positives, and the F1-score is their harmonic mean. These metrics are essential for evaluating fairness and robustness in non-iid data regimes [25,26,27]. From a systems standpoint, we measure the **end-to-end delay**, defined as the time taken from local computation (including encryption) through network transmission and aggregation to the final global model update. This is evaluated for both plaintext and encrypted FL to capture the latency overhead of HE [28]. We also report **INC network overhead**, broken down into *client-side encryption time* and *server-side aggregation time*, to analyze the scalability trade-offs of decentralized aggregation mechanisms. Finally, we report **energy consumption per round**, as derived from the AMR-GNN’s reward function, to evaluate energy efficiency—a critical factor for resource-constrained IoT and WSN nodes [27].

This collection of evaluation metrics provides a holistic understanding of our system’s trade-offs across accuracy, robustness, responsiveness, and energy efficiency in encrypted FL with INC support.

### 6.3. Simulation Results

**Figure 2:**
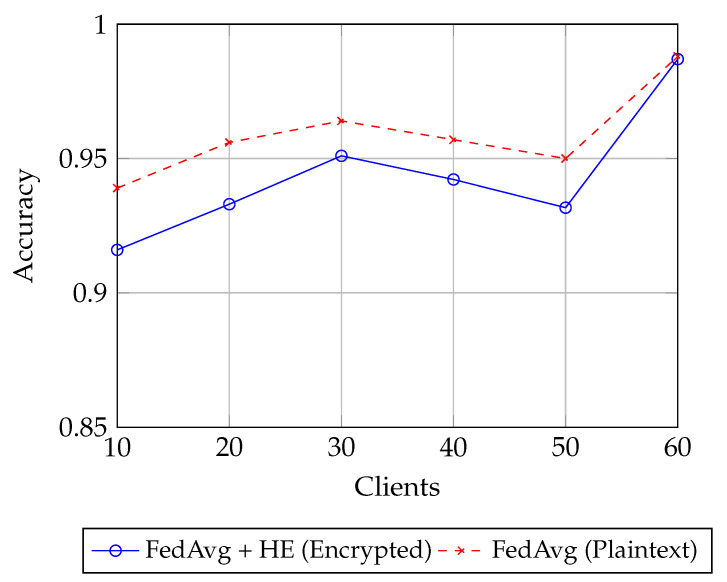
Accuracy of federated learning models under plaintext and encrypted settings across different numbers of clients (10–60).

With the above configuration in place, we next examine how model performance scales with the number of clients. As shown in Figure 2, both plaintext and encrypted FL maintain consistently high accuracy across different client counts, demonstrating that encryption does not significantly hinder model performance. At lower scales (10–20 clients), the plaintext model holds a small edge, reaching 0.95 accuracy compared to 0.92–0.93 for the encrypted setting. This gap narrows as the number of clients increases: by 30 clients, the encrypted model achieves 0.95, closely tracking the plaintext curve at 0.965. In the mid-range (40–50 clients), plaintext retains a slight advantage, fluctuating around 0.955–0.96, whereas the encrypted model stabilizes just below at 0.94–0.945. Importantly, at 60 clients both methods converge to nearly identical performance (0.99), indicating that with sufficient participation, the benefits of increased data diversity outweigh the minor computational losses introduced by encrypted training. The overall trend highlights two points: (i) encryption overhead produces only marginal differences, primarily visible at moderate client counts, and (ii) scalability in the number of participants strengthens accuracy in both cases, ultimately closing the performance gap. These findings reinforce that secure, privacy-preserving FL can scale effectively without sacrificing predictive utility.

**Figure 3:**
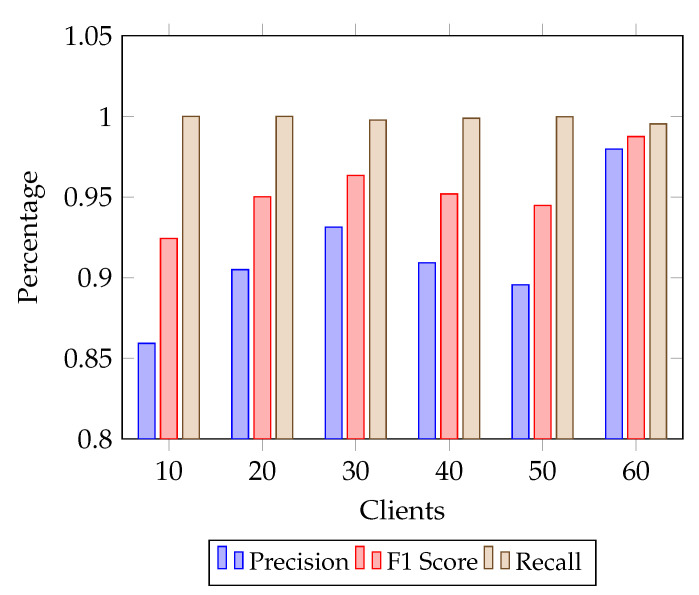
Precision, F1-score, and Recall of the encrypted FL model across different numbers of clients (10–60).

In Figure 3, the encrypted FL model demonstrates strong and balanced performance across precision, recall, and F1-score as the number of participating clients increases. Recall remains nearly perfect (1.0) throughout all client counts, underscoring the model’s ability to consistently identify true positives even under encryption. Precision starts at a modest level (0.85 at 10 clients), showing that with fewer clients the model is slightly more prone to false positives. However, it improves steadily as client participation increases, climbing above 0.90 at 30 clients and reaching 0.97 at 60 clients. This upward trend highlights the positive effect of greater data diversity on reducing misclassifications. The F1-score follows a similar trajectory to precision, beginning around 0.92 at 10 clients, stabilizing in the 0.94–0.95 range from 20 to 50 clients, and peaking near 0.97 at 60 clients. The combined picture indicates that, while recall is already saturated at high levels, precision and F1-score benefit most from scaling the number of clients. This balance shows that encryption introduces no measurable penalty on fine-grained classification performance, and in fact, larger federations enhance predictive reliability by minimizing false positives without sacrificing sensitivity.

**Figure 4:**
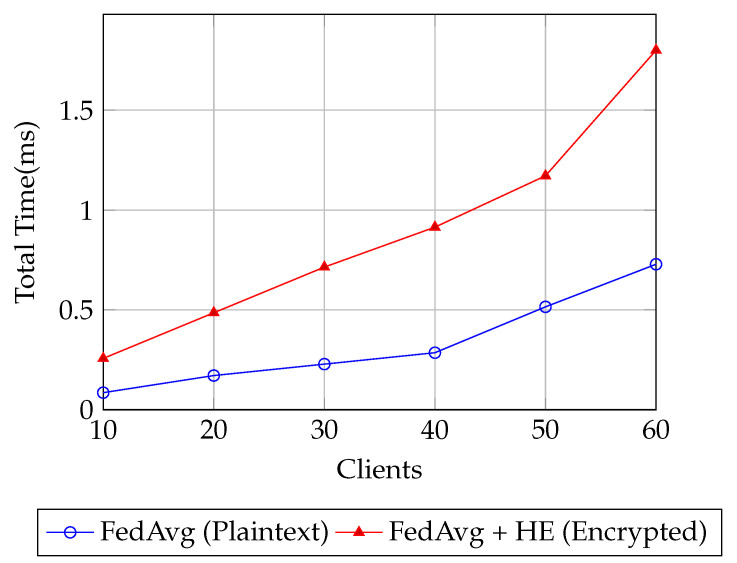
End-to-end delay of plaintext and encrypted FL across different client counts (10–60).

In Figure 4, the end-to-end delay for encrypted FL is consistently higher than plaintext due to the computational overhead of secure encryption operations and ciphertext aggregation. At smaller scales, this gap is modest: with 10 clients, plaintext incurs only 0.1 ms of delay compared to 0.25 ms for encryption. As the number of clients grows, both curves rise, but the encrypted system scales more sharply. For instance, at 30 clients, plaintext reaches 0.25 ms while encrypted is already at 0.7 ms; by 50 clients, the gap widens further to 0.5 ms versus 1.2 ms. At the largest federation tested (60 clients), plaintext delay is 0.7 ms, whereas encryption peaks at 1.8 ms. Despite this relative difference, the absolute delays remain well within the sub-2 ms range, which is practical for real-time or near real-time applications. These results emphasize the trade-off between privacy and latency: encryption adds measurable delay, but the increase is bounded and does not undermine the responsiveness of the system. This confirms that secure FL can be deployed at scale without critically compromising efficiency.

**Figure 5:**
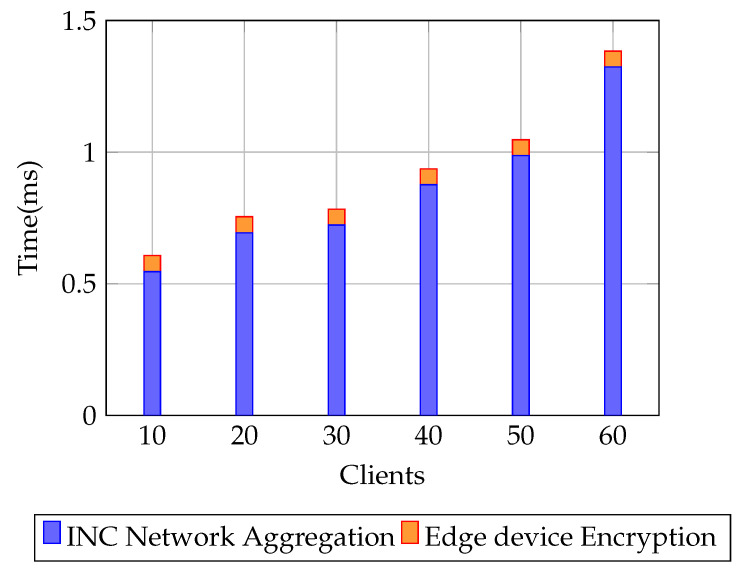
INC network overhead across client counts (10–60), decomposed into server-side aggregation and edge device encryption.

In Figure 5, the breakdown of network overhead clearly shows that server-side INC aggregation dominates the total cost, while edge device encryption adds only a minimal fraction. At lower participation levels (10–20 clients), the aggregation overhead is 0.55–0.7 ms, compared to 0.05–0.07 ms for encryption. As the client pool grows, the aggregation cost increases almost linearly, rising to 0.9 ms at 40 clients and 1.3 ms at 60 clients. The encryption overhead remains consistently small across all cases, increasing only marginally with scale, from 0.05 ms at 10 clients to 0.08 ms at 60 clients. Overall, the combined overhead remains modest, with total time just under 1.4 ms at the largest federation. These results emphasize two important points: (i) encryption on edge devices is lightweight and does not burden clients, and (ii) aggregation costs scale predictably with the number of clients but remain manageable in the millisecond range. Together, this division of overhead demonstrates that encrypted INC aggregation can scale efficiently for large federations while preserving client-side efficiency.

**Figure 6:**
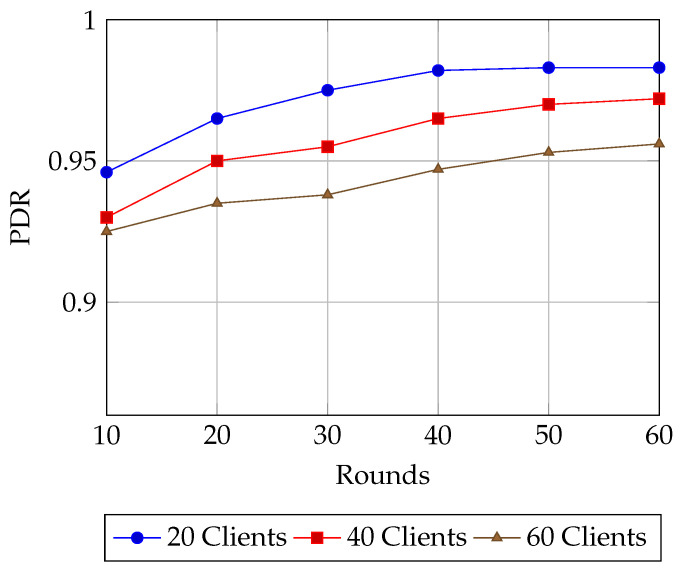
Packet Delivery Ratio (PDR) across training rounds for federations of 20, 40, and 60 clients.

In Figure 6, the Packet Delivery Ratio (PDR) exhibits steady improvement with increasing training rounds across all client configurations. For 20 clients, the system achieves rapid gains, surpassing 0.95 by round 20 and converging near 0.98 after 50 rounds, which reflects highly reliable delivery under lighter network load. With 40 clients, the growth trajectory is similar but slightly slower, with PDR reaching 0.97 by round 60, showing that the model adapts well to increased client participation. In the heaviest case of 60 clients, PDR starts lower (0.93 at round 10) and improves more gradually, stabilizing around 0.955 by the final rounds. This behavior indicates that higher client densities introduce additional communication overhead and routing complexity, yet the system maintains consistently high delivery rates. The narrow gap between lighter and heavier client cases highlights the effectiveness of the GNN-assisted in-network aggregation, which ensures robust and scalable packet transmission. Even at 60 clients, PDR remains well above 0.95, confirming the reliability of the framework in supporting encrypted update propagation under diverse loads.

**Figure 7:**
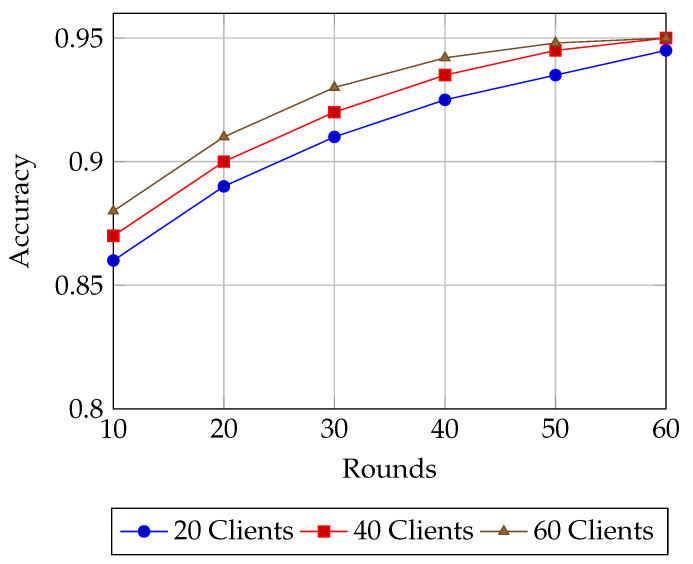
Accuracy of the GNN routing policy across training rounds for federations of 20, 40, and 60 clients.

In Figure 7, the GNN routing policy demonstrates steady and progressive accuracy gains across training rounds for all client configurations, converging near 0.95 by round 60. At the outset (round 10), accuracy starts at 0.86 for 20 clients, 0.87 for 40 clients, and 0.88 for 60 clients, showing that larger client groups provide an initial advantage due to richer path diversity and greater supervision. Over successive rounds, all three curves rise smoothly without oscillation: by round 30, accuracy has climbed to 0.91 for 20 clients, 0.92 for 40 clients, and 0.93 for 60 clients. At round 40, the 60-client setting leads with 0.94, while the 40-client and 20-client cases follow closely at 0.93 and 0.92, respectively. After 50 rounds, the gap between the configurations narrows considerably, with all three curves converging toward 0.95 by round 60 and the residual difference shrinking to less than half a percentage point. This monotonic improvement highlights the stability of the learning process, indicating that the AMR-GNN effectively generalizes its routing decisions without overfitting. Combined with the consistently high PDR shown in Figure 6, these results confirm that the proposed approach scales gracefully, maintaining accuracy and reliability as the client population grows.

**Figure 8:**
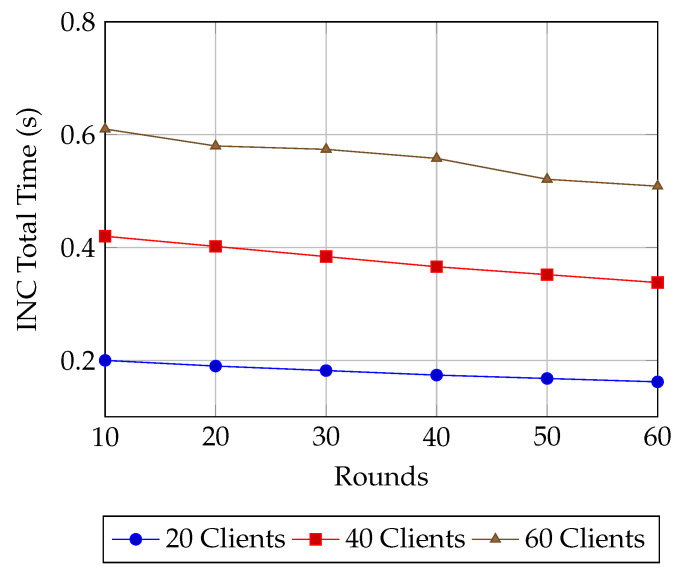
Total INC computation time across training rounds for 20, 40, and 60 clients.

In Figure 8, the total time for in-network computation (INC) steadily decreases with training rounds across all client configurations, confirming both the robustness and scalability of the proposed approach. For the 20-client case, the latency starts at approximately 0.20 s in round 10 and gradually drops to around 0.16 s by round 60, reflecting consistently efficient performance under light system load. In the 40-client scenario, the total time reduces from 0.42 s at round 10 to 0.34 s by round 60, showing that the system adapts well to medium-scale deployments. Even under the heaviest configuration with 60 clients, latency decreases from 0.61 s to 0.51 s over the same rounds, highlighting that the aggregation framework maintains efficiency despite increased communication and computation demands. The downward trend across all curves indicates that the GNN-assisted policy not only stabilizes but actively optimizes routing and aggregation strategies with training progression. This sustained reduction in latency underscores the system’s ability to scale effectively while delivering lower end-to-end overhead as learning matures.

**Figure 9:**
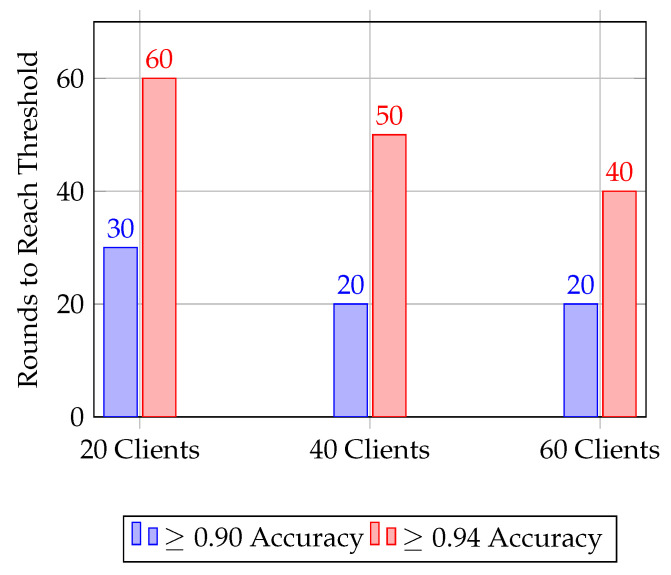
AMR-GNN convergence speed: rounds needed to reach 0.90 and 0.94 accuracy for different client scales.

The convergence characteristics observed in Figure 9 demonstrate the effectiveness of the proposed AMR-GNN in adapting to varying client scales. As the number of participating clients increases, the model converges in fewer rounds, indicating that the distributed learning process benefits from greater diversity in client updates. This outcome can be attributed to the algorithm’s design, wherein the adaptive reward function balances accuracy gains with energy and stability considerations, allowing the GNN to integrate heterogeneous routing information more efficiently. The reduced disparity between the rounds required to achieve moderate and high accuracy thresholds further underscores the robustness of AMR-GNN: once the model begins to converge, it maintains steady improvements without significant oscillations. Such behavior suggests that the algorithm avoids overfitting to smaller client distributions and instead leverages the broader participation to regularize learning, leading to faster and more consistent convergence. These findings highlight the suitability of AMR-GNN for large-scale, resource-constrained sensor networks, where both convergence speed and stability are critical.

**Figure 10:**
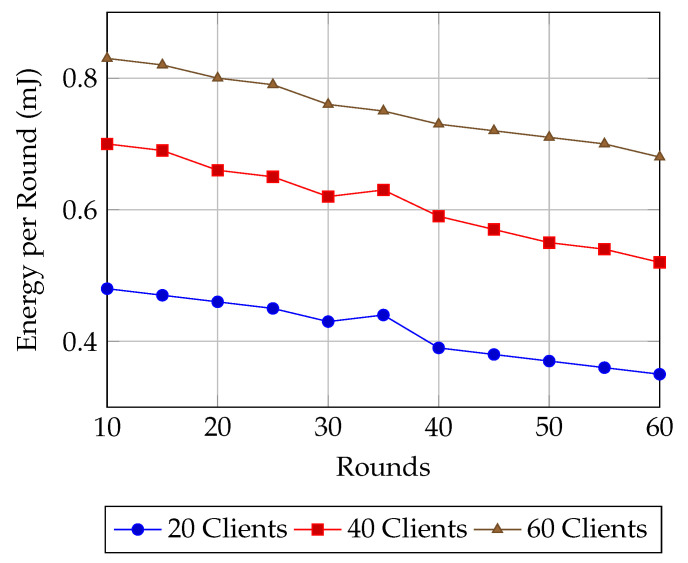
Energy per round from the AMR-GNN reward.

Finally, the energy consumption trends in Figure 10 highlight how AMR-GNN effectively balances routing accuracy with system efficiency. Across all client scales, energy per round exhibits a steady decline as training progresses, demonstrating that the reward mechanism successfully penalizes unnecessary overhead while reinforcing energy-aware routing decisions. The reduction is more pronounced for smaller client groups, where fewer nodes lead to more direct aggregation and lower communication costs. In contrast, larger client scales inherently consume more energy due to higher coordination and aggregation demands, yet even in these cases, the downward slope indicates that the algorithm learns to streamline resource usage over time. Importantly, the smooth decline without sharp fluctuations suggests that AMR-GNN avoids unstable routing oscillations, converging toward energy-optimal behaviors as the policy matures. This consistent reduction in per-round energy, coupled with the strong accuracy gains shown earlier, underscores the dual advantage of AMR-GNN: it achieves high-quality learning while progressively lowering system overhead, making it particularly suitable for deployment in resource-constrained sensor networks.

These results highlight two key strengths of our method. First, the steady downward trend across all cases reflects stable optimization without oscillations or divergence. Second, the sharper relative gains observed at larger client scales indicate that our design not only tolerates but actively benefits from richer supervision and higher aggregation demand. Together, these findings confirm that the proposed framework sustains efficiency while scaling to larger populations, ensuring tractable INC processing times and validating its suitability for real-world, high-density deployments.

## 7. Conclusions

In this work, we introduced EAH-FL, a novel framework that integrates HE with in-network aggregation to enable secure, low-latency federated learning in healthcare IoT environments. Unlike existing approaches that focus either on efficiency through INC or on privacy through encryption, EAH-FL unifies both dimensions into a single deployable architecture. By offloading cryptographic tasks to trusted edge devices and leveraging programmable switches for ciphertext aggregation, our design ensures that sensitive health updates remain encrypted end-to-end while achieving communication efficiency and scalability.

Through extensive simulations, we demonstrated that EAH-FL achieves competitive predictive performance compared to plaintext federated learning, with accuracy and F1-scores maintained across different client scales. At the same time, the framework supports high packet delivery ratios (PDR) and rapid GNN convergence for routing, confirming that encrypted updates can be transmitted reliably even in large-scale deployments. The latency and overhead results further highlight the practicality of our design: while encryption adds measurable cost, end-to-end delay remains within real-time bounds, and edge-side cryptographic operations contribute only marginally to total overhead. These findings underscore the significance of EAH-FL in mitigating the longstanding trade-off between privacy and efficiency in federated healthcare. The framework closes the gap between secure but computationally heavy HE-based solutions and efficient but plaintext INC-based systems, laying the foundation for real-world deployment of scalable and responsive health monitoring infrastructures that preserve privacy.

### 7.1. Limitations

Despite these promising results, several limitations of this study should be acknowledged. First, our evaluation relies primarily on simulation-based experiments. While simulations allow for controlled analysis of scalability, latency, and reliability, they inevitably abstract away certain real-world complexities such as device heterogeneity, unpredictable wireless interference, adversarial conditions, and fluctuating network traffic. A direct deployment on physical healthcare IoT testbeds would provide more conclusive evidence of robustness in practice.

Second, the proposed architecture assumes the presence of *trusted edge devices* that can perform encryption and partial aggregation securely. This assumption may not hold in adversarial environments where edge devices themselves can be compromised. Developing mechanisms for dynamic trust management or fully decentralized cryptographic validation remains an open challenge.

Finally, although the evaluation demonstrates the feasibility of secure aggregation and routing with EAH-FL, the analysis does not yet cover scenarios involving heterogeneous models, asynchronous federated learning updates, or cross-institutional collaborations. These aspects are critical for healthcare, where data arrives at different frequencies and institutional boundaries introduce additional privacy constraints.

### 7.2. Future Work

These limitations naturally open several avenues for future research. A key direction is the **deployment of EAH-FL on real-world healthcare IoT testbeds**, integrating wearable sensors, edge devices, and programmable switches under realistic network conditions. Such validation would provide deeper insights into system behavior under practical constraints and user mobility patterns.

Another promising line of work involves **advancing the cryptographic layer**. Extending the framework to multi-key HE would allow multiple stakeholders (e.g., hospitals, clinics, and research institutions) to collaborate without relying on a single key authority, thereby strengthening security guarantees. Similarly, adaptive key management and key rotation strategies can be investigated to ensure long-term security in dynamic IoT environments.

Lastly, future work should consider integrating **hardware accelerators for HE** and exploring hybrid approaches where only privacy-critical computations are encrypted, while less sensitive operations are performed in plaintext. This hybridization could strike an even better balance between computational efficiency and privacy preservation.

By addressing these open challenges, EAH-FL can evolve from a proof-of-concept framework into a deployable infrastructure for secure and efficient federated healthcare, providing both immediate practical benefits and long-term research opportunities. 

## Figures and Tables

**Table 1:** Summary of Related Works (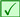 = addressed, 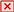 = not addressed, 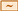 = partially addressed).

Authors	INC	HE	FL	Res. Constr.	Latency	End-to-End Enc.	Scalability
Su et al. [11]	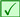	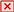	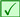	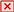	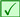	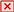	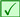
Xia et al. [12]	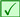	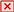	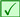	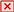	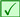	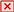	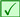
Ji et al. [18]	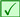	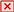	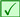	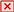	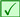	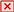	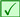
Zang et al. [13]	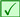	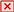	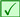	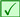	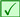	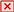	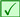
Choi et al. [4]	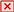	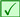	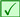	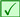	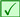	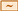	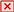
Yang et al. [2]	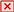	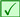	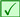	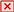	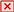	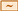	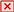
Shen et al. [5]	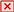	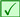	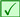	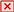	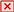	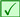	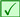
Walskaar et al. [6]	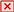	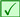	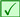	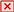	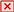	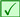	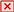
Naresh and Varma [17]	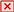	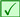	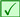	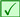	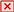	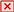	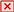
Firdaus et al. [7]	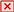	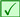	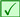	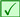	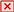	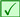	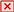
Lessage et al. [21]	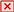	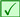	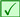	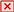	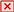	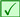	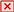
Hijazi et al. [23]	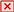	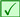	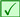	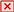	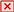	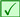	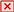
Jin et al. [22]	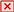	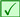	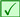	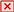	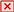	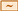	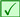
Gu et al. [20]	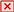	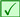	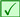	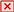	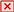	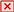	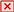
Lee et al. [3]	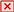	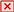	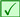	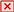	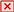	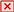	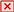
Aziz et al. [16]	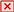	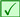	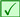	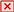	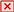	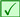	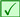
Mao et al. [8]	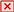	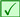	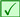	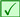	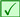	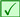	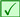
Gowri et al. [9]	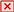	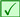	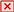	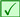	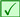	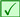	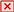
Khan et al. [10]	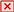	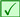	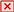	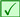	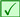	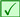	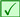
Caruccio et al. [14]	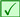	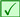	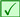	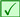	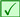	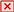	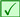
Albshaier et al. [15]	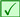	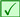	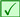	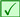	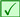	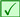	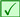
**This Work (EAH-FL)**	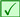	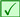	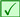	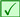	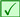	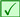	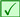

## Data Availability

The source code for the proposed framework and experiments is publicly available at: https://github.com/rahulkavati/fl_simulation (accessed on 30 September 2025).

## Abbreviations

The following abbreviations are used in this manuscript:

HE	Homomorphic Encryption
INC	In-Network Computing
CKKS	Cheon–Kim–Kim–Song (Homomorphic Encryption Scheme)
GNN	Graph Neural Network
AMRGNN	Adaptive multi-relational Routing Graph Neural Network
IoT	Internet of Things
HIPAA	Health Insurance Portability and Accountability Act
GDPR	General Data Protection Regulation
FL	Federated Learning
FHE	Fully Homomorphic Encryption
